# The structure of XIAP BIR2: understanding the selectivity of the BIR domains

**DOI:** 10.1107/S0907444913016284

**Published:** 2013-08-15

**Authors:** Christine Lukacs, Charles Belunis, Robert Crowther, Waleed Danho, Lin Gao, Barry Goggin, Cheryl A. Janson, Shirley Li, Stacy Remiszewski, Andrew Schutt, Manish K. Thakur, Saroj K. Singh, Srinivasan Swaminathan, Rajat Pandey, Rajiv Tyagi, Ramachandraiah Gosu, Ajith V. Kamath, Andreas Kuglstatter

**Affiliations:** a Discovery Technologies, Hoffmann-La Roche, 340 Kingsland Street, Nutley, NJ 07110, USA; b Medicinal Chemistry, Hoffmann-La Roche, 340 Kingsland Street, Nutley, NJ 07110, USA; c Discovery Oncology, Hoffmann-La Roche, 340 Kingsland Street, Nutley, NJ 07110, USA; d Structural Biology, Jubilant Biosys Ltd, Bangalore, India

**Keywords:** apoptosis, XIAP, BIR domains, caspases, extrinsic pathway, inhibitor of apoptosis, peptide complex, SMAC, AVPI

## Abstract

XIAP, a member of the inhibitor of apoptosis family of proteins, is a critical regulator of apoptosis. Inhibition of the BIR domain–caspase interaction is a promising approach towards treating cancer. Previous work has been directed towards inhibiting the BIR3–caspase-9 interaction, which blocks the intrinsic apoptotic pathway; selectively inhibiting the BIR2–caspase-3 interaction would also block the extrinsic pathway. The BIR2 domain of XIAP has successfully been crystallized; peptides and small-molecule inhibitors can be soaked into these crystals, which diffract to high resolution. Here, the BIR2 apo crystal structure and the structures of five BIR2–tetrapeptide complexes are described. The structural flexibility observed on comparing these structures, along with a comparison with XIAP BIR3, affords an understanding of the structural elements that drive selectivity between BIR2 and BIR3 and which can be used to design BIR2-selective inhibitors.

## Abstract 2

The high-resolution crystal structures of apo and peptide-bound XIAP BIR2 are presented and compared with BIR3 structures to understand their selectivity. This crystal system can be used to determine the structures of BIR2–inhibitor complexes.

## 1. Introduction   

Apoptosis can be triggered by initiation of either the intrinsic (mitochondrial) or the extrinsic (death-receptor-mediated) pathway. The intrinsic pathway can be activated by growth-serum withdrawal, radiation, DNA damage or other stress signals, causing a change in the outer membrane potential and permeability of the mitochondria (Fulda & Debatin, 2006 ▶). These changes allow the release of apogenic factors such as cytochrome *c* and second mitochondria-derived activator of caspase (Smac) into the cytosol, where cytochrome *c* can bind apoptosis protease-activating factor (Apaf1) and induce formation of the apoptosome. This in turn leads to the activation of caspase-9 and the subsequent downstream activation of caspase-3 and caspase-7 (executioner caspases).

The extrinsic pathway is initiated by the activation of membrane-associated death receptors of the TNF receptor superfamily by their respective ligands. Activation of these receptors leads to the formation of the receptor-associated FADD (Fas-associated death domain) complex and subsequent cleavage of caspase-8 and caspase-10. These processed caspases then cleave and activate caspase-3 and caspase-7 (Ashkenazi, 2008 ▶). This point represents the convergence of the intrinsic and extrinsic pathways, and the inevitable cleavage of downstream substrates that leads to cell death.

XIAP (X-linked inhibitor of apoptosis) directly inhibits the upstream caspase-9 and the downstream caspase-3 and caspase-7, and therefore controls critical apoptotic checkpoints (Holcik & Korneluk, 2001 ▶). The XIAP protein consists of several domains, including three zinc-containing BIR (baculovirus IAP repeat) domains (BIR1, BIR2 and BIR3) and a C-terminal RING domain. Although there is high homology among the BIR domains across the IAP family, they have different functions and specificities. The XIAP BIR2 domain and the linker region between the BIR1 and BIR2 domains are both required for the inhibition of activated caspase-3 and caspase-7 (Riedl *et al.*, 2001 ▶; Suzuki *et al.*, 2001 ▶; Huang *et al.*, 2003 ▶). The linker region blocks the active sites of these caspases, while the N-terminal regions of the partially processed caspase-3 and caspase-7 bind the BIR2 domain in a peptide-binding groove that accommodates the four N-­terminal residues (Huang *et al.*, 2001 ▶, 2003 ▶). The XIAP BIR3 domain targets and inhibits caspase-9 by binding monomeric caspase-9 and preventing its dimerization and activation (Shiozaki *et al.*, 2003 ▶; Scott *et al.*, 2005 ▶). Smac also binds in the same groove of XIAP through its N-terminal IAP-binding motif, AVPI, and associates primarily with the BIR3 domain (Srinivasula *et al.*, 2001 ▶), although it also has affinity for the BIR2 domain (Vaux & Silke, 2003 ▶; Samuel *et al.*, 2006 ▶). Smac is a dimer in solution, and it can bind both the BIR2 and BIR3 domains of XIAP simultaneously to prevent XIAP from binding and inhibiting caspase-3, caspase-7 and caspase-9 (Du *et al.*, 2000 ▶; Varfolomeev *et al.*, 2007 ▶).

Both BIR domains are important differential regulators of XIAP function and represent separate specific targets for therapeutic intervention. The XIAP BIR3 domain is a critical determinant for the inhibition of the mitochondrial (intrinsic) pathway owing to its association with caspase-9, making it a target for treating resistance to standard chemotherapy or radiation therapies. BIR3 antagonists have been shown to enhance the response *in vitro* of both chemotherapy and TRAIL/DR5 agonists (Bockbrader *et al.*, 2005 ▶) by direct activation of the initiator caspase-9 (Deveraux *et al.*, 1998 ▶). Whether or not a BIR2-selective antagonist that inhibits the XIAP-dependent repression of the downstream executioner caspases caspase-3 and caspase-7 can be efficacious either alone or in combination with known therapies remains to be seen. Unfortunately, to date there are no known potent and highly selective BIR2 antagonists; only weak polyphenyl urea compounds (Wu *et al.*, 2003 ▶; Schimmer *et al.*, 2004 ▶) and, more recently, Smac mimetics which show only moderate selectivity (González-López *et al.*, 2011 ▶) have been reported.

Crystal structures of the BIR3 domain of XIAP in complex with peptides, full-length binding partners and Smac mimetics have been described, and there are numerous inhibitors that show excellent selectivity for the BIR3 domain and cIAP over BIR2, primarily from the laboratory of Shaomeng Wang and from Genentech (comprehensively reviewed by Flygare & Fairbrother, 2010 ▶; Wang, 2011 ▶). Structure-aided drug design has been used in the design of these BIR3 inhibitors; a crystal structure of BIR2 could similarly guide the design of BIR2-selective inhibitors. However, the BIR2 domain has been more reluctant to crystallize. The apo structure has been solved by NMR (Sun *et al.*, 1999 ▶), and the only crystal structure showing the full XIAP BIR2 domain is in complex with caspase-3 (Riedl *et al.*, 2001 ▶). The major interactions described between BIR2 and caspase-3 are not through the AVPI binding groove but rather are at other sites. However, the AVPI binding site is occupied by the N-terminus of caspase-3 from a symmetry-related molecule, so it does not represent a true apo structure. Furthermore, this crystal form is unlikely to be useful for investigating the binding of small-molecule inhibitors. Numerous other BIR family members have also been crystallized: apo forms of XIAP BIR1, XIAP BIR2 (described here), cIAP1 BIR1, DIAP1 BIR2 and survivin, and peptide-bound forms of XIAP BIR2 (described here), XIAP mBIR3, ILP2, cIAP1 BIR3, cIAP1 BIR2, MLIAP, an MLIAP/XIAP chimera, NAIP, DIAP1 BIR1 and DIAP1 BIR2 (Herman *et al.*, 2009 ▶; Du *et al.*, 2012 ▶; Kulathila *et al.*, 2009 ▶; Riedl *et al.*, 2001 ▶; Huang *et al.*, 2001 ▶; Zobel *et al.*, 2006 ▶; Liu *et al.*, 2000 ▶; Wu *et al.*, 2000 ▶, 2001 ▶; Franklin *et al.*, 2003 ▶; Yan *et al.*, 2004 ▶; Nikolovska-Coleska *et al.*, 2004 ▶, 2008 ▶; Vucic *et al.*, 2005 ▶; Wist *et al.*, 2007 ▶). As with the XIAP BIR2–caspase-3 structure, several of these were not intentionally crystallized with peptide, but the N-terminus of a second protein molecule was found to bind in the peptide-binding groove.

We have successfully determined the crystal structure of the BIR2 domain of XIAP in its apo form. This crystal form can be used for soaking peptides and small molecules into the peptide-binding groove. Furthermore, these crystals diffract to better than 1.5 Å resolution. In this paper, we present the apo structure and five peptide-complex structures, and compare the structures of BIR2 and BIR3 in order to understand their different peptide-preference profiles and the selectivity of known inhibitors.

## 2. Materials and methods   

### 2.1. Cloning, expression and purification   

Methods for the cloning, purification and crystallization of XIAP BIR2 were initially determined at Jubilant Biosys Ltd. His6-TEV-hBIR2(152–236;C202A,C213G) was cloned into pET28a between *Nde*I and *Xho*I restriction sites. The final sequence was MGSSHHHHHHSSGLVPRGSHMENLYFQG-hBIR2(152–236;C202A,C213G), with TEV protease cleavage leaving a G before the start of the BIR2 sequence. The protein was expressed in *Escherichia coli* Rosetta2(DE3)pLysS cells using 2×YT medium. The cells were grown at 310 K until the OD_600_ reached 0.6, at which point 0.5 m*M* IPTG was added and the temperature was lowered to 289 K. The cells were harvested after an overnight growth period and then lysed in 10 ml 25 m*M* Tris pH 7.5, 100 m*M* NaCl, 5 m*M* imidazole, 0.5 m*M* TCEP, 5% glycerol, 10 µ*M* ZnCl_2_ per gram. 1 ml of protease-cocktail inhibitor was added per 100 ml and the suspension was sonicated and then cleared by centrifugation. The protein was purified *via* Ni–NTA affinity chromatography (Qiagen) using the same buffer as described above, with the imidazole concentration adjusted to 40 m*M* for washing and to 500 m*M* for elution. The eluted protein was concentrated and digested overnight with TEV protease against 2 l of the same buffer (without imidazole) in order to remove the His tag. The cleaved protein was further purified *via* size-exclusion chromatography (Superdex 75 gel filtration). The protein was concentrated to 5–20 mg ml^−1^ in a final buffer consisting of 25 m*M* Tris pH 7.5, 100 m*M* NaCl, 1 m*M* TCEP, 5% glycerol, 10 µ*M* ZnCl_2_.

### 2.2. BIR2 and BIR3 TR-FRET assay   

10 n*M* 6×histidine-tagged BIR2 (amino acids 124–240) or BIR3 (amino acids 241–356) domain of the XIAP protein was mixed with 20 n*M* of the peptide AVPIAQKSEK-(∊-biotin)-OH in a 1:2 ratio with TFA in the presence of 50 m*M* Tris–HCl pH 7.5, 100 m*M* NaCl, 1 m*M* dithiothreitol (DTT), 0.1 mg ml^−1^ bovine serum albumin (BSA). Following a 45 min incubation at 310 K, europium streptavidin and allophycocyanin-conjugated antihistidine antibody were added to final concentrations of 1.5 and 15 n*M*, respectively. Time-resolved fluorescence resonance energy transfer (TR-FRET) signals were measured 1 h later at room temperature. Compound potency was assessed at ten serially diluted concentrations. The percentage of inhibition at each concentration was determined to generate an IC_50_ value for each compound. Compound *K*_i_ values were calculated from the IC_50_ values using the Cheng and Prusoff equation (Cheng & Prusoff, 1973 ▶) for competitive inhibitors: *K*_i_ = IC_50_/(1 + [S]/*K*_m_), where [S] represents the substrate concentration. For target–ligand interactions, ligand *K*_d_ values are substituted. AVPIAQKSEK *K*_d_ values of 2.5 and 0.5 µ*M* for BIR2 and BIR3, respectively, were used.

### 2.3. Peptide synthesis   

Peptides were synthesized by solid-phase peptide synthesis (SPPS) using microwave-assisted peptide synthesis (Liberty peptide synthesizer; CEM Corporation, Matthews, North Carolina, USA). The crude peptides were dissolved in a minimum amount of water and acetonitrile and purified on a Shimadzu LC-8A system by high-performance liquid chromatography (HPLC) on a reverse-phase C18 column (50 × 250 mm, 300 *A* ^0^, 10 µm). Peptide was eluted using a 2–70% gradient of buffer *B* over 70 min (buffer *A*, 0.1% TFA in H_2_O; buffer *B*, 0.1% TFA in acetonitrile) at a flow rate of 60 ml min^−1^. UV detection was set at 220/280 nm. The fractions containing the products were separated and their purity was judged on a Shimadzu LC-10AT analytical system using a reverse-phase Pursuit C18 column (4.6 × 50 mm) at a flow rate of 2.5 ml min^−1^ in a gradient of 2–70% buffer *B* over 10 min (buffer *A*, 0.1% TFA in H_2_O; buffer *B*, 0.1% TFA in aceto­nitrile). Fractions judged to be of high purity were pooled and lyophilized.

### 2.4. Crystallization, data collection and structure refinement   

BIR2 protein at 5–20 mg ml^−1^ in final buffer was crystallized in sitting-drop format using a reservoir consisting of 1.7–1.9 *M* ammonium sulfate, 125 m*M* bis-tris propane pH 7.0 and drops consisting of equal volumes of protein solution and reservoir solution, typically 0.5 + 0.5 µl. Bipyramidal crystals appeared in 2–3 d and continued to grow for several days at room temperature (0.1–0.3 mm final size).

Peptides were prepared for soaking by dissolving them in DMSO to 20–50 m*M* and were then diluted to 2 m*M* in 1.8 *M* ammonium sulfate, 0.125 *M* bis-tris propane pH 7.0. The crystals were soaked with peptide solution for 3 d, although 1 d would be sufficient for these soluble peptides. Longer soaking times may be required for weaker and/or less soluble ligands. After soaking for 3 d, the crystals were swished through a cryosolution consisting of 75–80%(*v*/*v*) reservoir and 20–25%(*v*/*v*) glycerol and were then plunged into liquid nitrogen. Data for the AMRV complex were collected using a Rigaku MicroMax-007 HF X-ray generator equipped with Osmic VariMax HR optics and a MAR345dtb detector. The data were reduced with *HKL*-2000 (Otwinowski & Minor, 1997 ▶). The structure was solved by molecular replacement with *MOLREP* from *CCP*4 (Winn *et al.*, 2011 ▶) using the BIR2 chain from PDB entry 1i3o, which is the structure of the BIR2–caspase-3 complex (Riedl *et al.*, 2001 ▶). Data for the apo structure and the other peptide complexes were collected on beamline X10SA at the Swiss Light Source, which is equipped with a PILATUS 6M detector. Data were reduced with *XDS* (Kabsch, 2010 ▶) and *SCALA* (Winn *et al.*, 2011 ▶).

Refinement was carried out primarily using *CCP*4 (Winn *et al.*, 2011 ▶), *CNX* (Accelrys) and *Coot* (Emsley & Cowtan, 2004 ▶). Tyr154 was modelled with an occupancy value of 0.49 to eliminate a steric clash with its symmetry mate. Data-collection and refinement statistics are shown in Table 1 ▶. Final coordinates have been deposited in the PDB as entries 4j3y for the apo structure, 4j45 for the BIR2–ATAA complex, 4j44 for BIR2–AIAV, 4j46 for BIR2-AVPI, 4j47 for BIR2–SVPI and 4j48 for BIR2–AMRV.

## 3. Results and discussion   

### 3.1. XIAP BIR2 crystallization   

Although many IAP domains have successfully been crystallized, XIAP BIR2 has resisted structure determination for many years. The design of BIR2-selective inhibitors would be greatly aided by the availability of a crystallization system that would allow the cocrystallization or soaking of small molecules. We have succeeded in producing such crystals using a construct that spans residues 152–236 of XIAP (cutting off the linker region) and includes the same two cysteine mutations, C202A and C213G, as were incorporated into the construct used for NMR studies (Sun *et al.*, 1999 ▶), presumably for improved behavior in solution. XIAP BIR2(152–236;C202A,C213G) crystallizes as a dimer, with the N-­terminus of molecule *A* occupying the Smac binding site of molecule *B*. However, this binding does not mimic the binding mode observed in the Smac, caspase or peptide complexes; rather, it is likely to be the key to driving the crystallization of the protein.

In the apo structure, the binding site of molecule *A* is unoccupied (although glycerol from the cryoprotectant is sometimes observed in the P1 pocket) and is available for the soaking in of peptides or small-molecule inhibitors. We found that the size, affinity and solubility of the peptide (or ligand) are important. For example, weaker or less soluble binding partners may require soaking times of up to two weeks to achieve adequate occupancy in the crystal. Also, since the binding groove is near a symmetry-related molecule, the peptide or ligand is near its crystallographic mate; thus, there is a limit to the size of the ligand that can occupy the site without destroying the crystal.

### 3.2. XIAP BIR2 apo structure   

**Figure 1:**
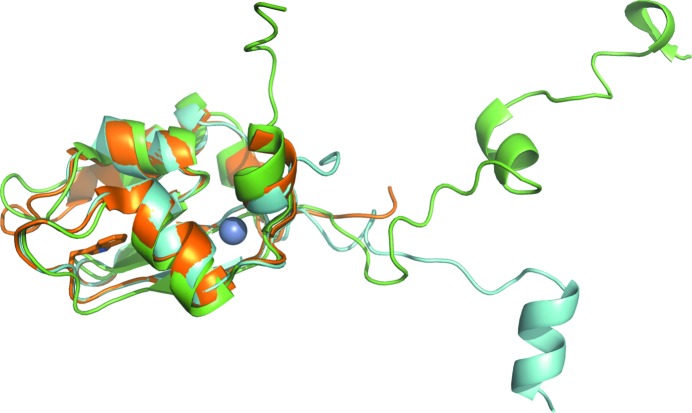
Overlay of the XIAP BIR2 crystal structure at 1.35 Å resolution (orange), the NMR structure (green; PDB entry 1c9q; Sun *et al.*, 1999 ▶) and the caspase-3-bound form (light blue; PDB entry 1i3o; Riedl *et al.*, 2001 ▶). The construct used in crystallization removes the flexible and variable preceding linker region as well as residues from the C-terminus. For the apo structure, only chain *A* is shown; in the other molecule the N-­terminus is disordered up to residue 159. The zinc is shown as a sphere and Trp210, which creates the floor of the binding groove, is shown in stick representation.

As has been reported previously for other BIR domains, the overall architecture of the BIR2 domain is a three-stranded antiparallel β-sheet surrounded by five α-helices, with a structurally important zinc residue coordinated by Cys200, Cys203, His220 and Cys227. Fig. 1 ▶ shows an overlay of our apo crystal structure, the apo structure determined by NMR (Sun *et al.*, 1999 ▶) and the caspase-bound form of XIAP BIR2 (Riedl *et al.*, 2001 ▶). The linker preceding the N-terminus of the domain (the connector between BIR1 and BIR2) varies in these different crystal structures, most notably in the caspase-bound form, in which the linker region makes important interactions with the caspase (Riedl *et al.*, 2001 ▶; Scott *et al.*, 2005 ▶); the linker region has also been shown to be functionally important in biochemical experiments (Shin *et al.*, 2005 ▶). The NMR structure also has a long flexible linker leading into the BIR2 domain. Because the N-terminus is clearly different for each system, and in order to obtain a stable domain for crystallization, this structurally variable region was removed from our BIR2 construct. On the other hand, the rest of the protein, including the peptide-binding site, of caspase-3-bound XIAP BIR2 and our apo structure is very similar.

### 3.3. AVPI, SVPI, ATAA, AIAV and AMRV bound to BIR2   

Franklin *et al.* (2003 ▶) and Sweeney *et al.* (2006 ▶) have published detailed analyses of the specificity profiles of BIR2 and BIR3. Sweeney and coworkers screened a combinatorial tetrapeptide library against both BIR2 and BIR3 in order to define the peptide-specificity profile for both domains. Their experiments show that while alanine is essentially unalterable in the P1 pocket of both (BIR3 can accommodate valine), the P2 through P4 pockets display strikingly different specificities; in general, BIR3 shows much stronger preferences than BIR2. For BIR3, there is a slight preference for lysine or arginine followed by small aliphatics in P2, while proline or arginine is strongly preferred in P3 and large hydrophobic residues, with a strong preference for phenylalanine, in P4. For BIR2, although the preferences for P2 through P4 are less strong, P2 seems to prefer valine/threonine/glutamine/arginine/tyrosine/isoleucine/serine. Alanine is somewhat preferred at P3, followed by arginine and lysine, while small residues (valine/alanine/glycine) predominate in P4.

Phage-display analysis by Franklin and coworkers showed a striking difference in the P3 and P4 sites: while BIR2 can accept larger residues in P3, BIR3 cannot. Inversely, BIR2 shows a strong preference for the aliphatic residues valine and isoleucine at P4, while BIR3 selected peptides with phenyl­alanine or tryptophan 88% of the time in P4. In contrast to the study of Sweeney and coworkers, Franklin and coworkers did not find arginine in the P3 position of any selected BIR3-binding peptides. Table 2 ▶ shows a summary of the combined findings.

We tested a series of peptide sequences in BIR2 and BIR3 inhibition assays (Table 3 ▶) and the results agree well with those of Franklin and Sweeney. Replacing the N-terminal alanine with a serine causes a loss of activity for both BIR2 and BIR3, although this is a conservative change. The crystal structures of BIRC1 and BIRC3 and XIAP BIR2–caspase-3 all have an N-terminal serine at the P1 position; the O^γ^ H atom is within hydrogen-bonding distance of the glutamine at the edge of the pocket (Herman *et al.*, 2009 ▶). Furthermore, one of the glycerol molecules that was observed in our initial apo structure overlays well with these serine residues.

**Figure 2:**
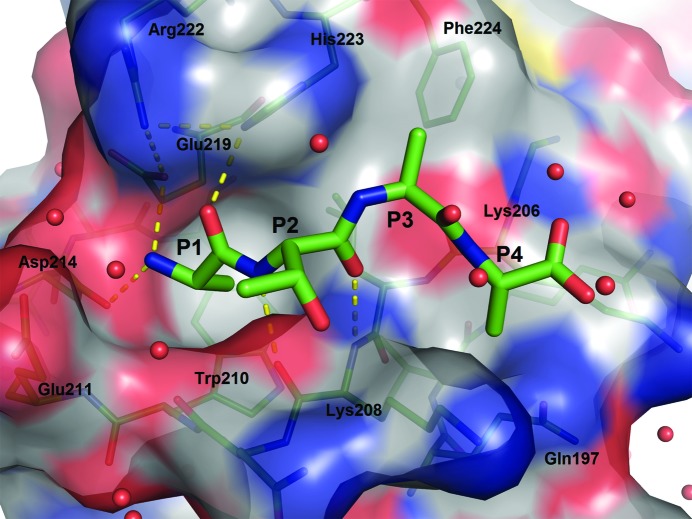
The ATAA tetrapeptide bound to the XIAP BIR2 domain, showing the protein surface as represented in *PyMOL*. Important residues are labeled. Hydrogen bonds are shown as yellow dashes.

The most potent tetrapeptide among those assayed against BIR2 is ATAA, which interestingly was inactive against BIR3 in our assay. In fact, all peptides with alanine at the P3 position were essentially inactive against BIR3. On the other hand, ARPI and AIPI, which fit the BIR3 preferential sequence pattern, are both very potent against BIR3 (*K*_i_ values of 0.12 and 0.08 µ*M*, respectively) but are much less potent against BIR2 (10.17 and 3.11 µ*M*, respectively). With the crystal structures of both BIR3 and BIR2 now available, one can rationalize these trends from a structural perspective. Therefore, the structures of XIAP BIR2 with five different peptides soaked in and bound to the Smac binding groove were also solved: ATAA, AVPI, SVPI, AIAV and AMRV. It is instructive to begin with an analysis of the simplest and most potent, ATAA, as shown in Fig. 2 ▶.

As in all described BIR-domain structures, the P1 alanine points to the face of Trp210, which creates the bottom face of the pocket. The N-terminal amino group is anchored by several hydrogen bonds to the surrounding acidic residues Asp214 and Glu219. Arg222, which is poorly resolved in the apo structure, is well resolved in the peptide-bound structure, bending in towards the binding groove to interact with Glu219 and His223. His223 in the apo structure was modelled so that the δ-NH can donate a hydrogen bond to the backbone of Glu219. However, when there is peptide bound it is equally valid to flip the side chain so that the δ-NH is pointing towards the P1 carbonyl. In BIR3, this residue is a tryptophan and the equivalent of Arg222 is a lysine, which cannot reach the peptide. Thus, from a structural perspective, BIR2 can perhaps better anchor the P1 peptide.

P2 makes standard backbone-to-backbone hydrogen-bond interactions with BIR2, and the side chain points towards solvent. The only interactions are hydrophobic ones with the side chains of Asn209 and Lys208, perhaps leading to the weak preference of BIR2 for hydrophobic residues at P2 (see the AMRV structure), but explaining the general lack of strong preferences for this residue.

The P3 alanine is rotated so as to sit along the hydrophobic surface of the protein, and the P4 alanine points into a small pocket created by the side chains of Lys206, Lys208 and Gln197, although Gln197 can rotate away to make a deeper pocket, as will be shown later. Finally, the P4 backbone NH donates a hydrogen bond to the backbone of Lys206.

**Figure 3:**
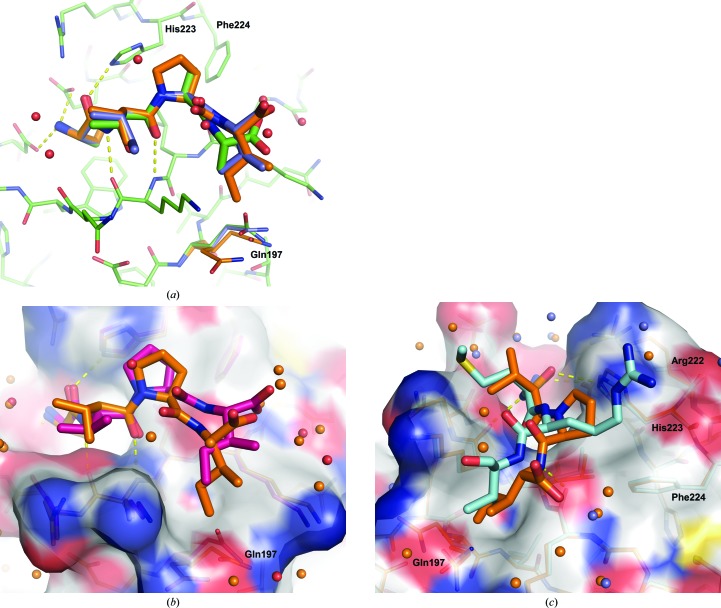
(*a*) Overlay of ATAA (green), SVPI (orange) and AIAV (blue). A combination of peptide movement and rotation of Gln197 allows the protein to accommodate the differently sized P4 residues. Gln197 was observed in two conformations in the SVPI structure. (*b*) Overlay of SVPI (orange) and AVPI (red). Instead of Gln197 moving away, the peptide is pushed out. (*c*) Overlay of SVPI (orange) and AMRV (blue). The arginine causes the backbone to shift enough that the hydrogen bond to the Leu207 backbone is lost.

Of the other four peptides, SVPI overlays most closely with ATAA (Fig. 3 ▶ *a*). Even with a serine at P1, the P1–P3 residues bind nearly identically. However, the P4 isoleucine is much larger than alanine, and even though Gln197 rotates away from the pocket (in fact, Gln197 has two alternate conformations in this structure), the larger pocket created by this movement is still not large enough to accommodate the isoleucine side chain without a shift in the backbone. The position of SVPI in BIR2 aligns well with most published BIR domain–AVPI structures. Next, AIAV binds identically to SVPI, but with an intermediate position observed for Gln197, which does not have to move as much to accommodate the valine as it does for the isoleucine. This series of structures highlights the flexibility of the protein and the value of having multiple structures for comparison.

**Figure 4:**
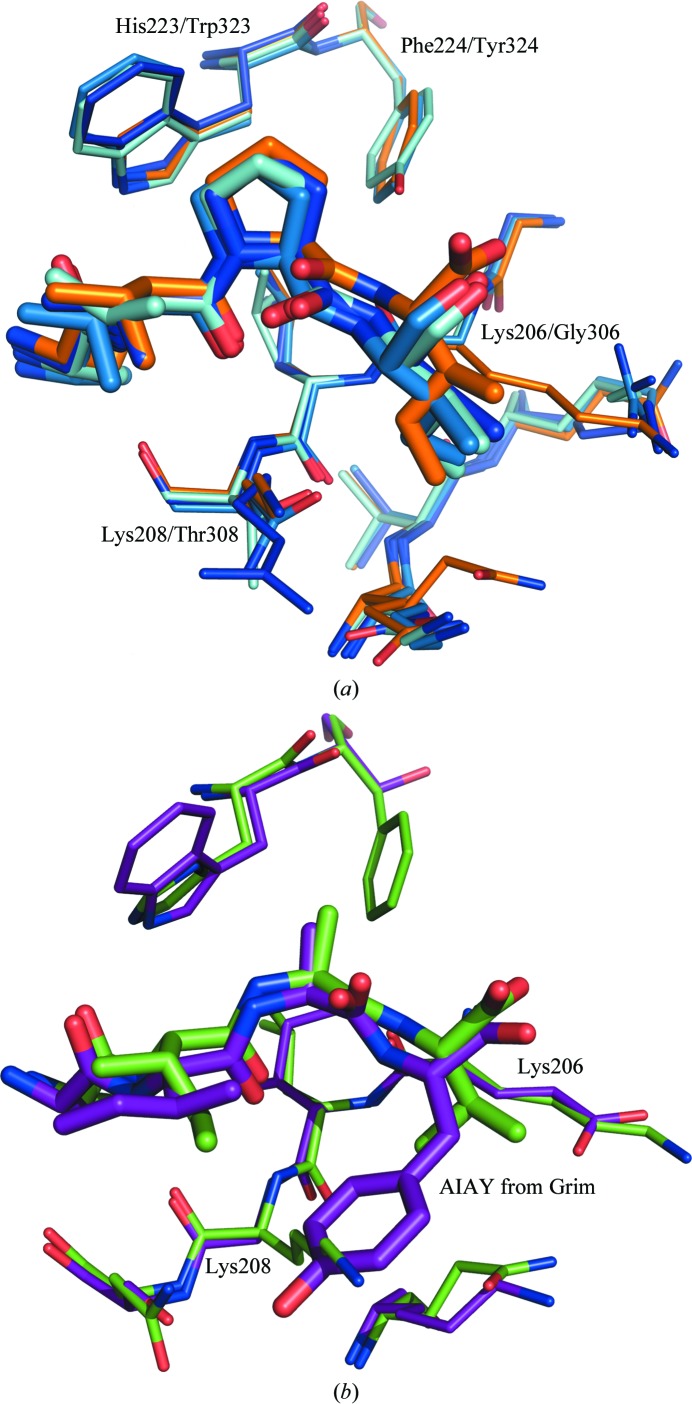
(*a*) Overlay of AVPI structures: XIAP BIR2–SVPI in orange, XIAP BIR3–AVPI in cyan (PDB entry 1g73; Wu *et al.*, 2000 ▶), MLIAP–AVPI in turquoise (PDB entry 1oxq; Franklin *et al.*, 2003 ▶) and CIAP1 BIR3–AVPI in dark blue (PDB entry 3d9u; Kulathila *et al.*, 2009 ▶). Note that Lys206 is a glycine in XIAP BIR3, MLIAP and CIAP BIR3 and this restricts the size of the pocket, so that P4 is buried less deeply. (*b*) Overlay of XIAP BIR2 with AIAV bound (green) and DIAP1 BIR1 with the Grim peptide bound (purple; AIAY from Grim shown; PDB entry 1seo). In this case, Lys208 is a glycine in DIAP1 BIR1, allowing the large tyrosine at P4 to fit.

It was assumed that AVPI would bind in a nearly identical manner to SVPI and that this structure could be used in the comparison of AVPI and SVPI to examine the effect of the alanine-to-serine substitution in the P1 pocket (SVPI is less potent than AVPI), but surprisingly the AVPI peptide takes on a different conformation (Fig. 3 ▶ *b*). The additional hydroxyl introduced by the serine substitution results in only a small shift in the side chains of His223 and Leu207 to allow the extra atom. Instead, the P4 pocket remains small, with Gln197 filling the pocket, and the isoleucine is forced away (Fig. 4 ▶ *a*). Owing to this different conformation, the P2 valine takes on a different rotamer in order to avoid a steric clash with the proline carbonyl.

Finally, the structure with a much more different peptide, AMRV, which contains a different combination of residues that impart selectivity for BIR2 over BIR3, was solved (Fig. 3 ▶ *c*). The methionine at P2 shows how the side chains of Asn209 and Lys208 can flank and provide a hydrophobic environment for a longer P2 side chain. Owing to the large arginine at P3, there must be a compensatory shift in the peptide backbone in order to allow the side chain room to fit, since the C^α^—C^β^ bond of the arginine lies flat against the protein (as was noted for ATAA). While the P4 valine only needs a shallow pocket, the P3 shift is large enough that the P4 NH to Leu207 backbone interaction is lost. Note that in the crystal structures of BIRC1 and BIRC3 (PDB entries 2vm5 and 2uvl) with bound SMRY/V peptides the P3 arginine is in a different conformation than in XIAP BIR2 (Herman *et al.*, 2009 ▶). Both of these proteins have a Trp at position 223 and a Phe at position 224, and the arginine must fit in between them.

### 3.4. Comparison of XIAP BIR2 and XIAP BIR3 structures   

As we and others have shown, there is a significant difference in the specificities of the various BIR domains for peptides (Franklin *et al.*, 2003 ▶; Sweeney *et al.*, 2006 ▶). Peptidomimetics based on Smac and targeting XIAP BIR3 have been reported which are much more potent against BIR3 and cIAP than BIR2 (Flygare & Fairbrother, 2010 ▶; Wang, 2011 ▶). With the crystal structure of BIR2 in hand, the structures of BIR2 and BIR3 were compared in order to understand these specificities from a structural perspective. Three important residue differences were identified, His223/Trp323 (numbering is for BIR2/BIR3), Phe224/Tyr324, and Lys206 + Lys208/Gly306 + Thr308, which play a role in BIR-domain specificity, some or all of which can be exploited to design in specificity of inhibitory peptides or small molecules.

BIR3 shows a strong preference for proline at the P3 position. One of the critical differences between the BIR2 and BIR3 domains is His223/Trp323. In BIR3, the large aromatic Trp323 is securely positioned; the indole N atom donates a hydrogen bond to the side-chain O atom of Gln319 (or the P1 carbonyl when peptide is bound) and one face of the indole makes hydrophobic interactions with the side chain of Lys322. The other face interacts with P3; in particular, the methylene H atoms of the proline C^δ^ interact favourably with the π system of the tryptophan. This interaction does not exist in BIR2, in which the smaller His223 seems to be more important for stabilizing the protein through hydrogen bonds or by interacting with the P1 peptide carbonyl.

The side chain of the P3 residue is also in proximity to the Phe224/Tyr324 side chain. Although a phenylalanine-to-tyrosine mutation is considered to be conservative, a significant polar and steric difference is introduced by the additional hydroxyl group. This puts further restrictions on the identity of P3: BIR2 can fit most side chains within the cleft between- His223 and Phe224, while BIR3 only allows smaller or more flexible side chains (no charges or large aromatics) to fit between Trp323 and Tyr324 (Sweeney *et al.*, 2006 ▶). Even alanine is not well tolerated by BIR3; the β carbon is pulled away from the tyrosine when it is part of the restricted cyclic environment of a P3 proline. Activated caspase-3, which has SGVD at the N-terminus, binds BIR2 but not BIR3. The same argument holds for a valine at P3: BIR3 cannot accommodate it in the more limited space between the tryptophan and tyrosine residues. The Phe224/Tyr324 difference not only changes the size and polarity of the environment of the P3 side chain, but it also adds another hydrogen-bond donor/acceptor to the binding pocket. One could imagine that under certain steric conditions the P4 backbone NH, or an appropriately placed hydrogen-bond donor in a small molecule, might interact with the tyrosine OH instead of the Gly306 backbone.

The P4 side chain points towards the protein. The shallow pocket that surrounds P4 is dramatically different between BIR2 and BIR3, specifically the residues Lys206/Gly306 and Lys208/Thr308. The two lysines in BIR2 plus Gln197 form a pocket in BIR2 that is about 8.5 Å wide and can nicely accommodate flat (aromatic) or hydrophobic side chains. The Gln197 can move away, resulting in a pocket that allows longer moieties in this pocket, as was shown in the P4 Ala→Val→Ile series. However, it is clear that Phe, Tyr and Trp would be too large to fit and would presumably be pushed out far enough to likely cause a loss in binding affinity. This also helps to explain why activated caspase-9, which has ATPF at the N-terminus, binds BIR3 more potently than BIR2. Conversely, the lysine–lysine pair places a limitation on where the P4 backbone can be in BIR2, and the backbone of the peptide or peptido­mimetic in many of the small-molecule inhibitors may need to shift to accommodate this restriction.

In BIR3, on the other hand, the lack of any side chain at the Gly306 position allows the P4 residue greater flexibility in its position and preference, allowing the backbone to maximize its interactions with the protein, picking up the interaction with Gly306 or possibly even Tyr324. It is clear from the overlay of the two structures that when P4 is a phenylalanine, as in AVPF, it would clash with residue 306 were it nonglycine. It is instead Lys297 and Lys299 in BIR3, which are deeper in this pocket, that terminate the binding site (Gln197 and Gln199 in BIR2). Speer and coworkers agree; in describing their heterodimeric bi-specific peptides, they generated a K206G mutant of BIR2 which restored the potency of BIR2 for AVPF (Speer, Cosimini *et al.* 2012 ▶). Interestingly, in DIAP BIR1 the corresponding residues are glycine and glutamate: the glutamate places a similar restriction on the position of the peptide as Lys206 in BIR2; however, the lack of a side chain on the other side allows DIAP to nicely accommodate even large residues such as tyrosine, as is observed in the DIAP–Grim(AIAY) structure (Chai *et al.*, 2003 ▶). Fig. 4 ▶(*a*) shows an overlay of SVPI in BIR2 with several BIR domain–AVPI structures, highlighting the Lys206 difference, and Fig. 4 ▶(*b*) shows an overlay of AIAV with DIAP1 BIR1 with AIAY bound, highlighting the Lys208 difference.

## 4. Conclusions   

We have successfully crystallized and solved the structure of XIAP BIR2 by itself for the first time and we have shown how this system can be used to soak peptides into the AVPI peptide-binding groove. We have shown in a series of peptide-bound structures that the protein is somewhat flexible and can adjust the conformation of the side chains in the binding groove to accommodate differences in the peptide. These results are consistent with the reported peptide-preference profiles for BIR2 and BIR3, and provide a structural basis for the binding-partner pairings of BIR2 with caspase-3 and of BIR3 with caspase-9.

Numerous groups have reported peptides and peptido­mimetics that show preferential binding for BIR3 and CIAP over BIR2. With a direct comparison of the two domains now available, it is clear that the differences in a few key residues can be taken advantage of to design compounds that preferentially bind to one domain over the other. In particular, it seems that the His/Trp + Phe/Tyr combination in the P3 area and the Lys/Gly + Lys/Thr combination which makes a dramatically different environment for the P4 residue are especially important to drive the differences in potency. The Smac mimetics and small-molecule inhibitors with published crystal structures all place aromatic rings into the P4 environment and are clearly not compatible with Lys206 (Wist *et al.*, 2007 ▶; Sun *et al.*, 2008 ▶; Cossu *et al.*, 2010 ▶). The challenge in designing BIR2-specific inhibitors is likely not to be in designing out the potency for BIR3, but in increasing the potency for BIR2.

## Supplementary Material

PDB reference: XIAP BIR2, apo, 4j3y

PDB reference: ATAA complex, 4j45

PDB reference: AIAV complex, 4j44

PDB reference: AVPI complex, 4j46

PDB reference: SVPI complex, 4j47

PDB reference: AMRV complex, 4j48

## Figures and Tables

**Table 1:** Data collection and refinement statistics Values in parentheses are for the highest resolution shell.

Structure (PDB code)	Apo (4j3y)	ATAA (4j45)	AIAV (4j44)	SVPI (4j47)	AVPI (4j46)	AMRV (4j48)
Data collection						
X-ray source	X10SA, SLS	X10SA, SLS	X10SA, SLS	X10SA, SLS	X10SA, SLS	Rigaku MicroMax-007 HF
Detector	PILATUS 6M	PILATUS 6M	PILATUS 6M	PILATUS 6M	PILATUS 6M	MAR345dtb
Space group	*I*422	*I*422	*I*422	*I*422	*I*422	*I*422
Unit-cell parameters ()	*a* = *b* = 74.5, *c* = 108.7	*a* = *b* = 74.5, *c* = 108.8	*a* = *b* = 74.9, *c* = 109.0	*a* = *b* = 74.5, *c* = 108.7	*a* = *b* = 74.8, *c* = 108.6	*a* = *b* = 74.8, *c* = 109.1
Resolution range ()	381.45	381.48	381.30	381.35	381.42	50.02.10
Total unique reflections	27428 (3921)	25875 (3703)	38399 (5528)	33922 (4861)	29457 (4248)	9105 (737)
Completeness (%)	99.9 (99.7)	99.9 (99.8)	100.0 (100.0)	100.0 (100.0)	100.0 (100.0)	97.0 (80.1)
Multiplicity	12.2 (12.1)	12.7 (13.1)	12.6 (12.8)	12.5 (12.4)	12.6 (13.1)	7.6 (2.8)
*I*/(*I*)	18.5 (4.8)	26.1 (5.2)	30.5 (5.7)	24.3 (5.5)	26.1 (5.7)	10.7 (1.9)
*R*_merge_	0.068 (0.450)	0.047 (0.479)	0.039 (0.451)	0.051 (0.470)	0.047 (0.476)	0.139 (0.427)
Refinement						
Total reflections in refinement	27428 (18787)	25875 (1801)	38399 (2659)	33921 (2311)	29456 (2040)	8678 (481)
Reflections in *R*_free_ set	1372 (109)	1344 (91)	2001 (151)	1774 (138)	1549 (111)	428 (27)
Final *R* factor/*R*_free_	0.185/0.203	0.174/0.216	0.174/0.193	0.175/0.194	0.173/0.199	0.186/0.253
No. of atoms						
Protein/Zn	1324/2	1304/2	1312/2	1307/2	1298/2	1306/2
Peptide	0	23	26	29	28	31
Waters	122	132	154	149	121	66
Average *B* factor (^2^)						
Protein/Zn	23.8/14.8	26.8/17.3	20.2/12.3	20.9/12.7	23.1/14.3	33.0/23.1
Peptide	n/a	34.6	19.4	19.6	22.3	35.6
Waters	34.4	38.9	33.0	33.3	34.0	36.3
R.m.s. deviations						
Bond lengths ()	0.016	0.016	0.013	0.014	0.015	0.016
Bond angles ()	1.63	1.69	1.52	1.57	1.63	1.62
Ramachandran plot						
Favored region (%)	98.7	99.4	99.4	98.7	98.7	97.5
Allowed region (%)	1.3	0.6	0.6	1.3	1.3	2.5
Outlier region (%)	0.0	0.0	0.0	0.0	0.0	0.0

**Table 2:** Amino-acid preferences in the binding grooves of BIR2 and BIR3 Data adapted from Franklin *et al.* (2003 ▶) and Sweeney *et al.* (2006 ▶).

	P1	P2	P3	P4
BIR2	Ala >> Ser	Smaller aromatic or Pro	Ala > Arg/Lys/Val	Val/Ala/Ile/Gly
BIR3	Ala >> Val	Lys/Arg > branched aliphatic	Pro >> Arg Ala	Phe/Tyr > Ile/Leu

**Table 3:** Inhibition constants for tetrapeptides against XIAP BIR2 and XIAP BIR3 The assay is a FRET-based assay measuring the displacement of an AVPI-derived peptide from the BIR domain (see [Sec sec2]2). Asterisks denote peptides which have been soaked into XIAP BIR2 crystals.

Tetrapeptide	BIR2 *K*_i_ (*M*)	BIR3 *K*_i_ (*M*)
ATAA*	1.70	>47
AVAV	1.87	26.08
AIAV*	1.87	28.2
ATAV	2.17	32.26
AMRV*	2.41	32.90
AVVV	2.54	
AIPI	3.11	0.08
AMRI	3.52	5.20
AVPI*	5.24	0.71
ARPI	10.17	0.12
SVPI*	12.02	4.62
SMRV	12.25	>47
SMPI	33.97	12.54
ARPR	>53	
